# Recurrent Giant Cell Tumor of the Lateral Condyle of the Distal Femur Managed by Repeated Extended Curettage and Bone Cementing: A Case Report

**DOI:** 10.7759/cureus.111051

**Published:** 2026-06-17

**Authors:** Sushil Mankar, Aditya A Patel, Rahul H Sakhare, Ronit P Dalvi, Aakarsh

**Affiliations:** 1 Orthopaedics and Traumatology, N. K. P. Salve Institute of Medical Sciences and Research Centre and Lata Mangeshkar Hospital, Nagpur, IND; 2 Orthopaedics, N. K. P. Salve Institute of Medical Sciences and Research Centre and Lata Mangeshkar Hospital, Nagpur, IND; 3 Orthopaedic Surgery, N. K. P. Salve Institute of Medical Sciences and Research Centre and Lata Mangeshkar Hospital, Nagpur, IND

**Keywords:** bone cement, distal femur tumor, extended curettage, giant cell tumor of bone, recurrent giant cell tumor

## Abstract

Giant cell tumor of the bone (GCTB) is a locally aggressive benign neoplasm with a significant propensity for local recurrence following intralesional surgery. Isolated involvement of the lateral condyle of the distal femur is uncommon. Extended intralesional curettage with chemical adjuvants and polymethylmethacrylate bone cementing is the accepted limb-salvage treatment. Management of recurrent disease, particularly with repeat joint-preserving surgery, and long-term outcomes beyond 10 years following revision procedures remain underreported in the literature. A 40-year-old male presented with a six-month history of right knee pain and difficulty walking. Radiography demonstrated a well-defined eccentric expansile lytic lesion with a soap-bubble appearance involving the lateral condyle of the right distal femur. Computed tomography confirmed cortical erosion and soft-tissue involvement. Fine-needle aspiration cytology yielded hemorrhagic fluid. Histopathology confirmed giant cell tumor. The patient underwent extended curettage, phenolization, and gentamicin-infused cementing in August 2012. Two years later, local recurrence was detected radiologically; repeat extended curettage and re-cementing were performed in September 2014. The patient remained disease-free at a 13-year follow-up (February 2026) with full knee range of motion, bilateral weight-bearing ambulation, and a structurally intact cement mantle on serial radiographic surveillance. This case proves that repeated curettage with phenolization and cementation is a viable option for limb salvage in cases of recurrent GCTB involving the distal femoral lateral condyle without radiographic spread. Thirteen years of tumor-free survival, functional integrity, and persistence of cement mantle after repeated procedures indicate the efficacy of the procedure, making it a preferable option to wide excision.

## Introduction

Giant cell tumor of the bone (GCTB) represents a locally aggressive benign primary bone tumor, accounting for around 4-5% of all primary bone tumors and 20% of all benign bone tumors [1]. This tumor occurs mainly in adults in the second to fourth decades of life. The distal femur is the most common anatomical location, being responsible for 25-30% of all cases [2]. The lateral condyle of the distal femur is a particularly rare anatomical localization of GCTB. GCTB is known to occur as an eccentric lytic epiphyseal lesion that may extend to the subjacent subchondral bone.

The limb-salvage procedure for accessible GCTB lesions involves extended curettage assisted with either phenol or hydrogen peroxide as chemotherapeutic agents together with bone cementing using polymethylmethacrylate (PMMA) [3,4]. The problem with this treatment modality is the high risk of recurrence, ranging from 10 to 40% in published studies [5,6]. Recurrence is common in the first two years after surgery and is linked to tumor grade, cortical perforation, and soft-tissue involvement. Management of recurrent GCTBs may involve curettage, extensive resection with reconstruction, or even administration of denosumab, with the latter remaining controversial.

There are very few follow-up results reported 10 years after surgical treatment for recurrent GCTB. We report an extremely rare case of recurrent GCTB involving the right lateral condyle of the distal femur, which was treated twice by extensive curettage, phenolization, and bone cementing. The patient remained free from recurrence for 13 years with a functional knee joint and intact bone cement throughout 12 years of imaging follow-up.

## Case presentation

A 40-year-old male presented in May 2012 with a six-month history of insidious-onset, gradually progressive pain over the right knee associated with difficulty in walking. He denied any history of significant trauma, fever, weight loss, or constitutional symptoms. There was no family history of bone tumors or malignancy. Systemic examination was unremarkable. Local examination of the right knee revealed mild swelling and tenderness localized to the lateral aspect of the right distal femur. There was no overlying skin erythema, local warmth, or palpable lymphadenopathy. There was complete knee range of motion. Distal neurovascular examination was normal. The contralateral limb showed no abnormality.

**Figure 1:**
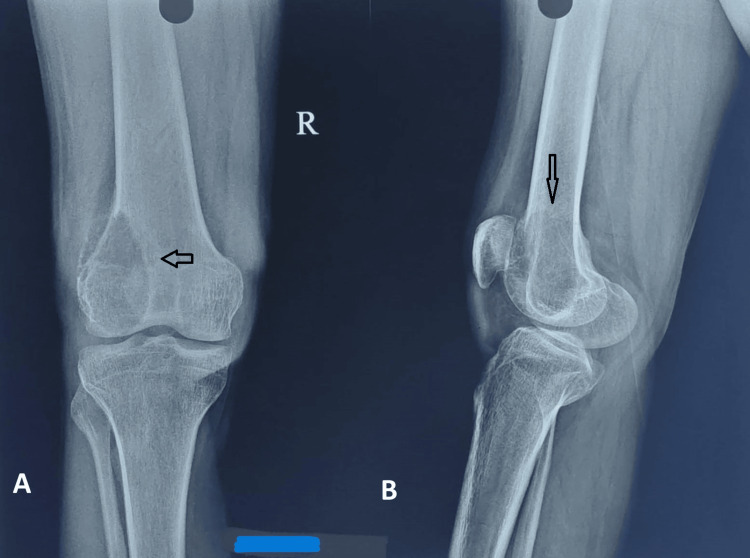
(A) Anteroposterior plain radiograph of the right knee joint showing a lytic lesion involving the right lateral condyle of the distal femur, with the arrow showing the margins of the lesion. (B) Lateral plain radiograph of the right knee joint, with the arrows showing the lesion in the right distal femur in both the viewss.

Plain radiography demonstrated a well-defined eccentric expansile lytic lesion at the metaphysis and epiphysis of the lateral condyle of the right distal femur, with a classic soap-bubble trabecular pattern. Adjacent cortical thinning was noted, with a lamellated periosteal reaction (Codman’s triangle) at the superior margin; no frank cortical breach was identified. There were no sclerotic margins, intramedullary calcification, or evidence of pathological fracture. The knee joint space was preserved (Figure 1).

**Figure 2:**
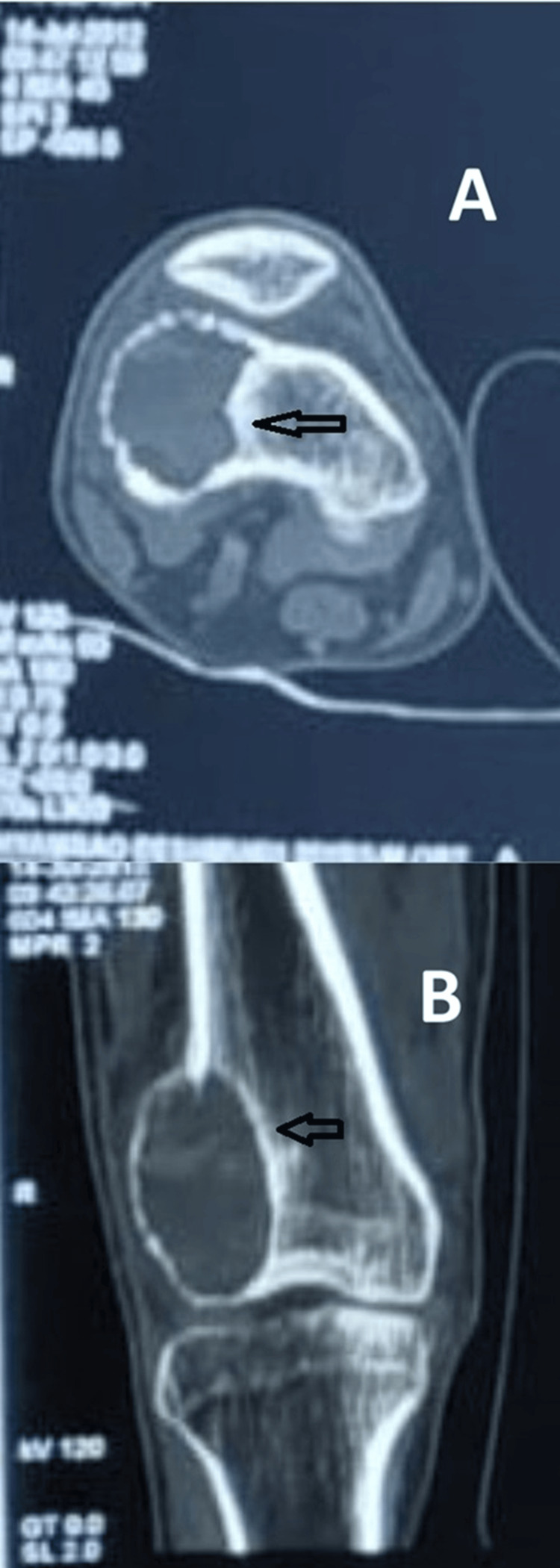
(A) Axial CT scan showing a lytic lesion centered in the right lateral condyle of femur. (B) Coronal CT scan showing cortical erosion in the right distal femur lateral condyle, with the arrow showing the medial margins of the lesion in both the views.

CT scan of the right knee confirmed an eccentric lytic lesion centered in the right lateral condyle with cortical erosion and soft-tissue component involvement. Three-dimensional reconstructions clearly delineated the full extent of the lesion and guided surgical planning (Figure 2).

**Figure 3:**
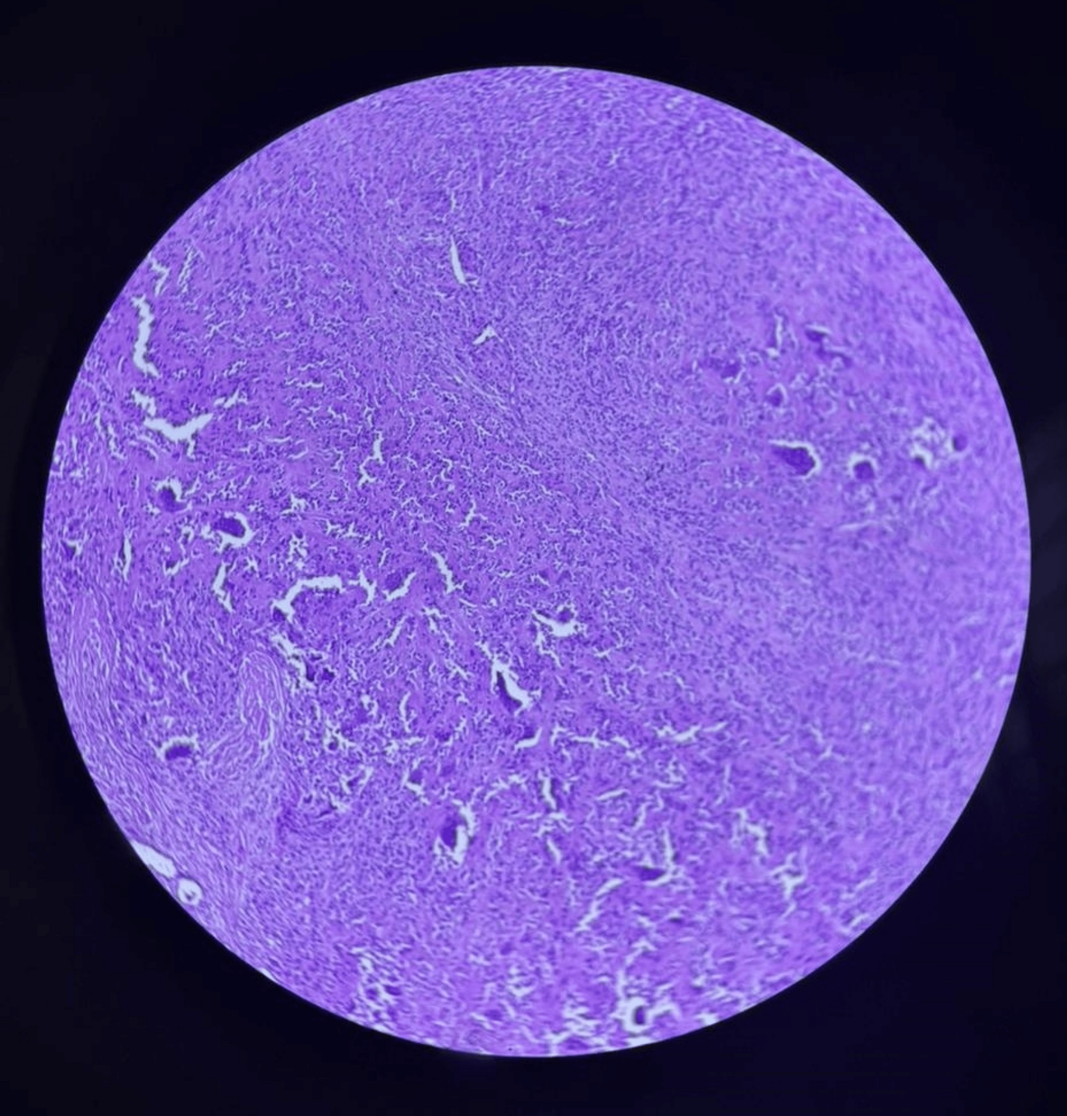
Histopathology images showing giant cell-rich area.

Fine-needle aspiration cytology (FNAC) yielded predominantly hemorrhagic fluid with few lymphocytes. Giant cells were not identified. Hemorrhagic fluid is a non-diagnostic result well-recognized in GCTB due to its intrinsically hemorrhagic and cystic components. Histopathological examination revealed uniformly distributed osteoclast-like multinucleated giant cells set in a mononuclear stromal background suggestive of giant cell tumor (Figure 3).

**Figure 4:**
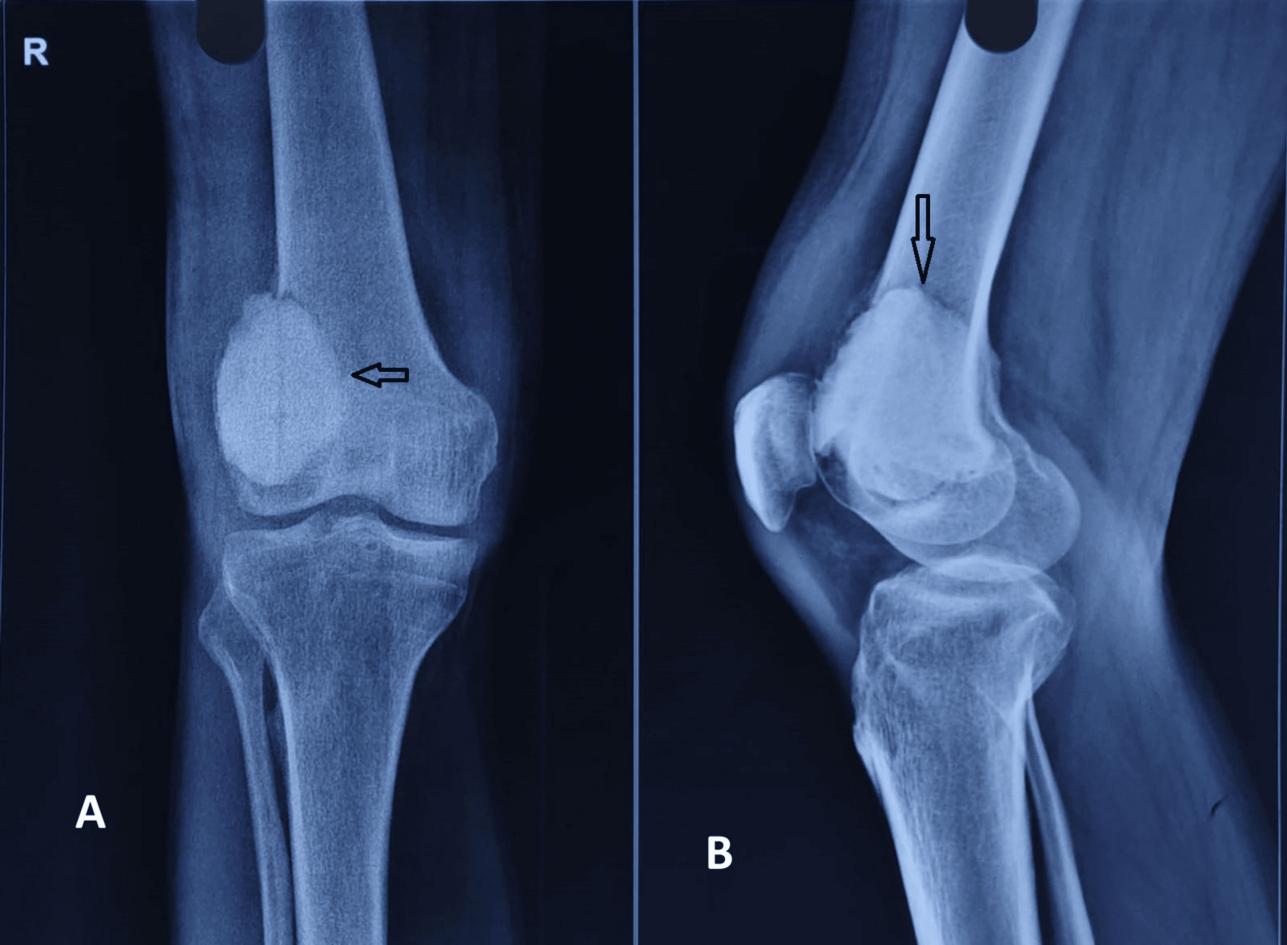
Postoperative (A) anteroposterior radiograph showing the cavity filled with bone cement in the right distal femur. (B) Lateral radiograph showing the intact cement mantle, with the arrows showing the well-demarcated margins of cement-filled cavity in both the views.

Following preoperative optimization, extended curettage of the lesion was performed using a high-speed burr, followed by adjuvant therapy with phenol. The cavity was thoroughly washed with normal saline, and then 40 g of gentamicin-infused bone cement was filled in the cavity (Figure 4).

**Figure 5:**
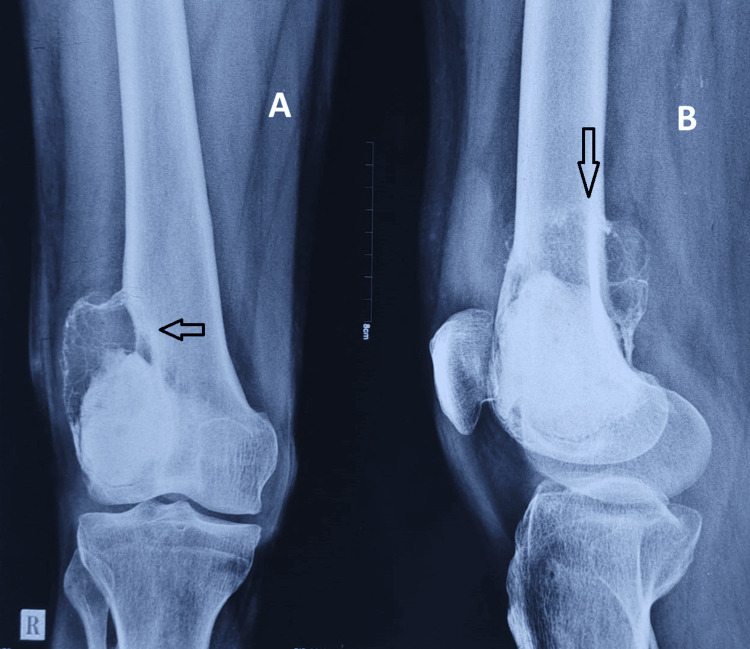
Two-year postoperative plain (A) anteroposterior radiograph showing lytic changes adjacent to the previously placed cement mantle. (B) Lateral view showing the margins of recurrent lytic changes, with the arrows showing the margins of new lytic changes in both views.

The patient remained asymptomatic for the next two years. Approximately 24 months after the index surgery, he presented with recurrence of pain over the right distal femur. Plain radiographs demonstrated new lytic changes adjacent to the previously placed cement mantle at the right lateral condyle, with evidence of abnormal bony growth consistent with local recurrence (Figure 5).

**Figure 6:**
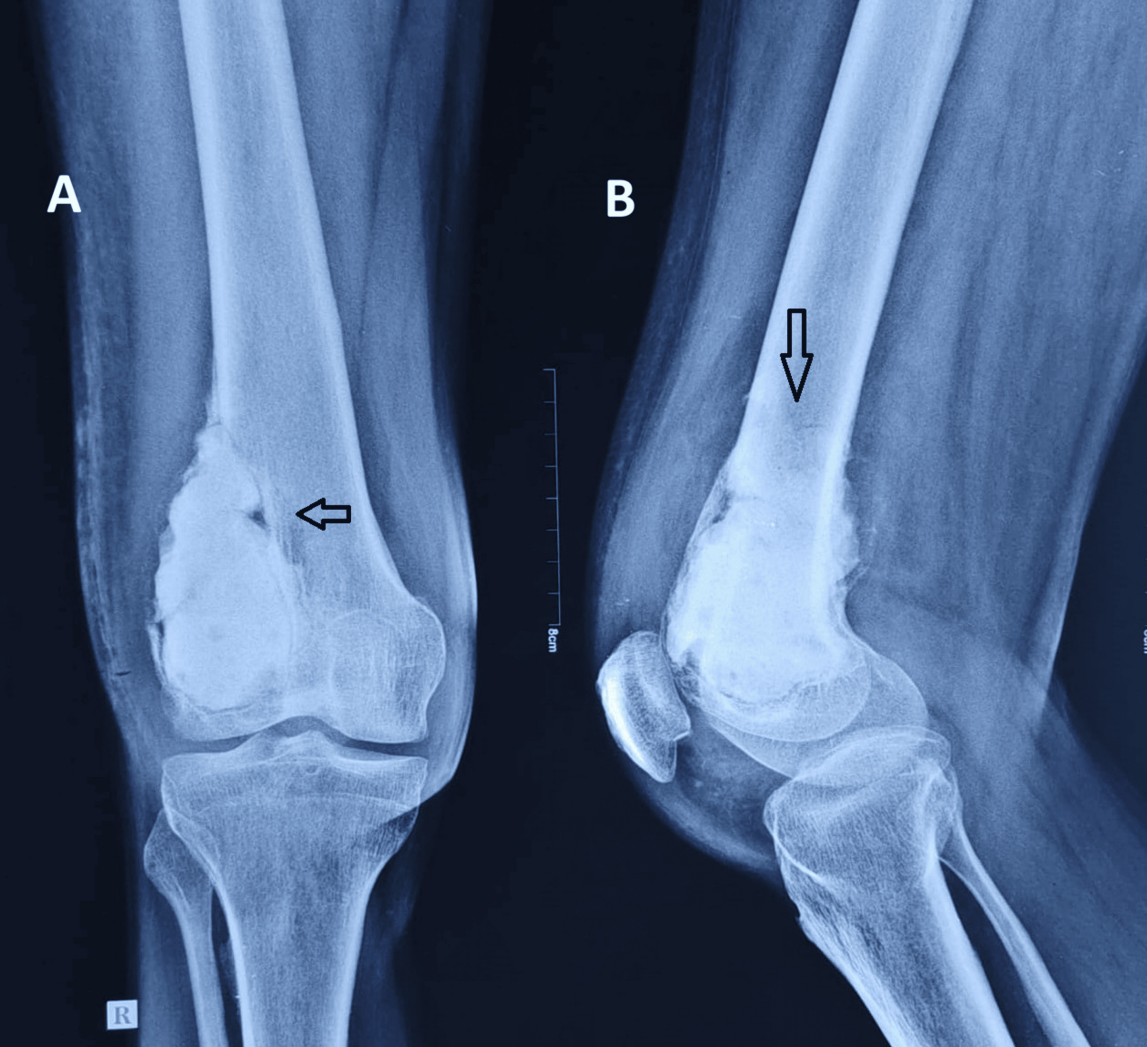
Two-year post-recurrence plain radiograph (A) anteroposterior view showing revision surgery with extended curettage of the cavity filled with bone cement. (B) Lateral view showing cavity filled with bone cement, with the arrows showing the margin of the cavity in both views.

The patient was recounselled regarding the recurrence and the available management options. After 24 months of the index surgery, the recurrent GCTB was excised, and extended curettage was performed, followed by phenolization of the cavity walls. Gentamicin bone cement (40 g) was re-applied (Figure 6).

**Figure 7:**
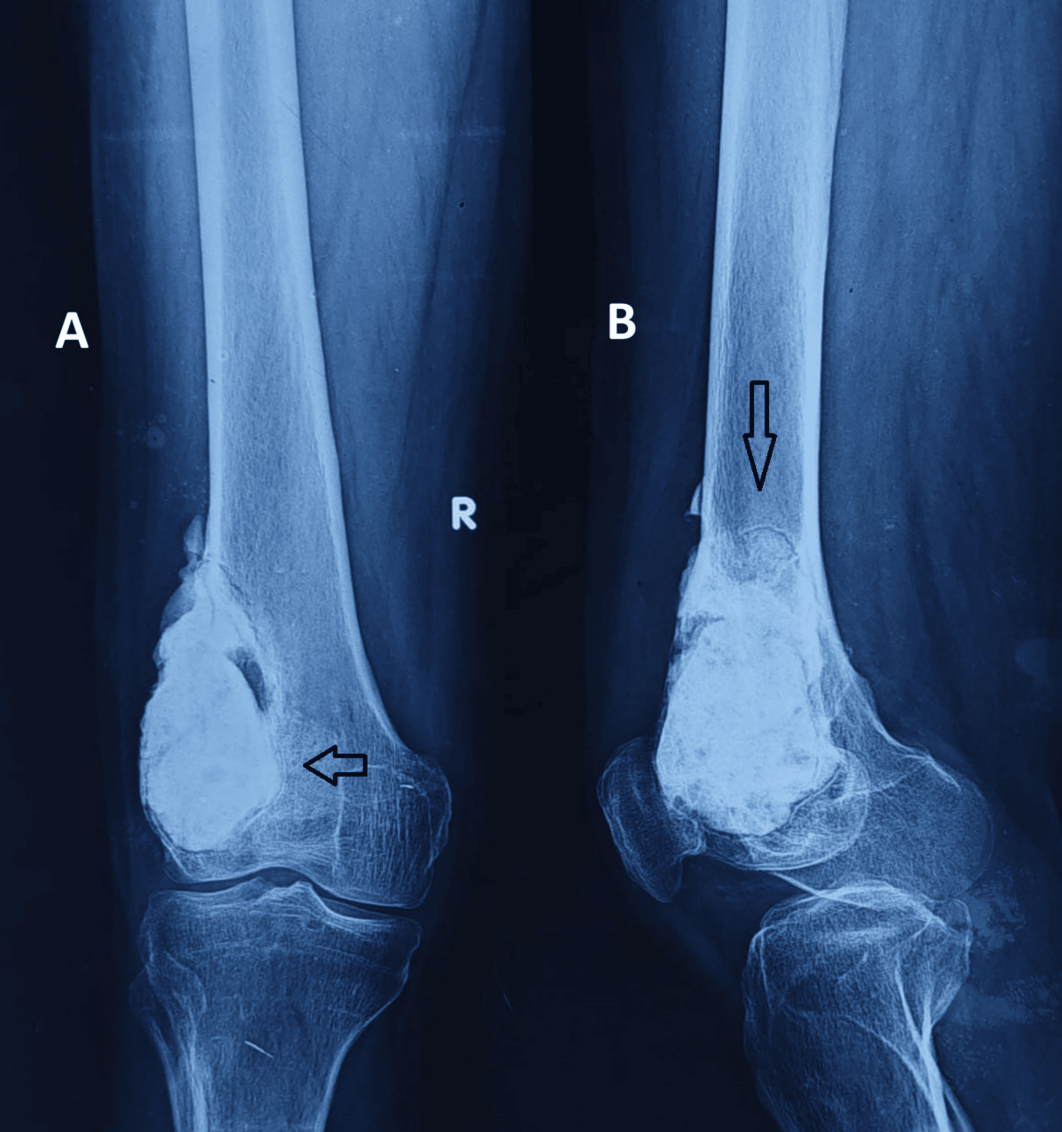
Three-year follow-up radiograph (A) anteroposterior view showing intact cement mantle and no lytic change. (B) Lateral view showing intact margins, with the arrrows showing margins of the intact filled cavity in both views.

The patient was followed at regular intervals post-revision surgery with clinical examination and plain radiography. At the three-year follow-up, plain radiograph revealed an intact cement mantle, and no lytic changes were seen with a normal joint space (Figure 7).

**Figure 8:**
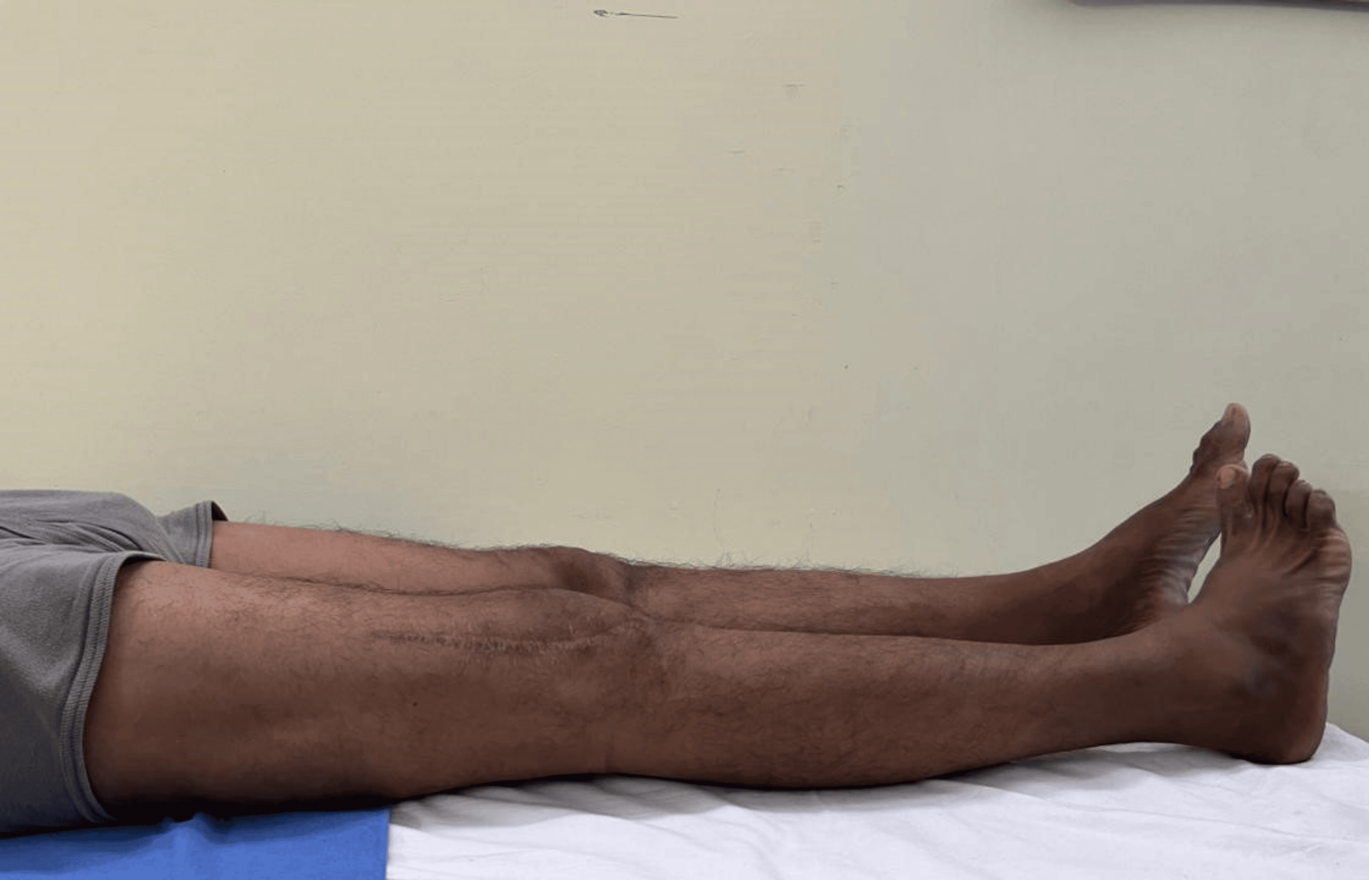
Clinical photograph showing full extension (12-year follow-up after revision surgery).

**Figure 9:**
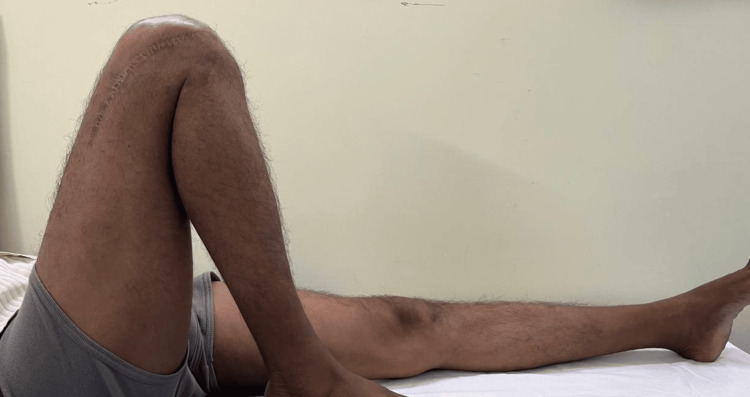
Clinical photograph showing full flexion (120°) (12-year follow-up after revision surgery).

During the latest follow-up 12 years after the revision surgery, the clinical examination of the right knee revealed full active extension (0°) (Figure 8), flexion up to 120° (Figure 9), no valgus or varus deformity, no joint line tenderness, no instability, and a healed lateral scar of 12 cm.

**Figure 10:**
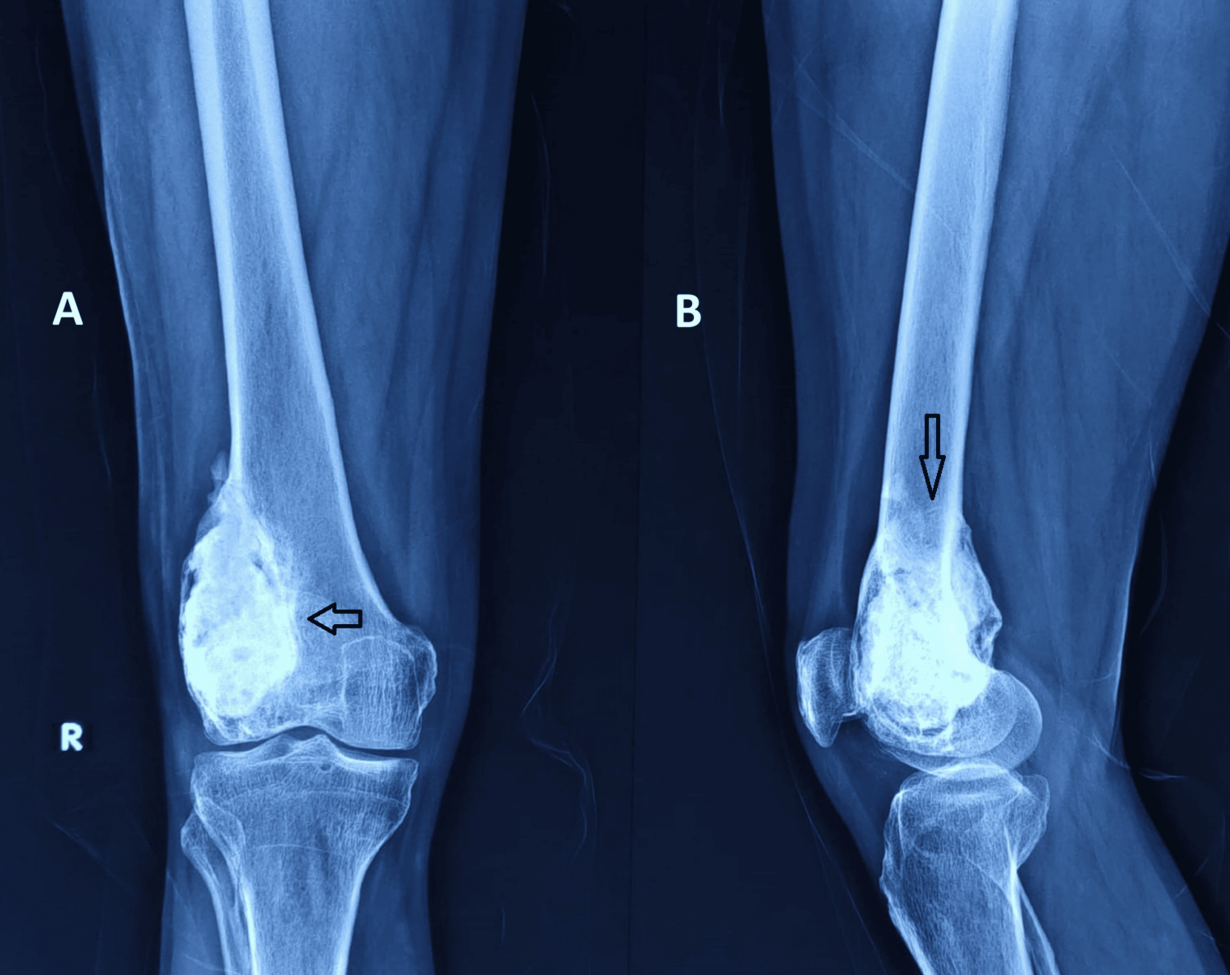
12-year follow-up post-recurrence surgery plain radiograph (A) anteroposterior view showing an intact cement mantle in the right lateral condyle without any lesions around and cement loosening/migration. (B) Lateral view showing mature bone growth, with the arrows showing mature bone growth in both views around the cement-filled cavity.

Radiologic evaluation (anteroposterior and lateral views) showed an intact cement mantle in the right lateral condyle without any lesions, cement loosening/migration, a normal joint space, and mature bone growth around the site (Figure 10). No signs of late local recurrence or degenerative joint disease were noted.

## Discussion

GCTB of the distal femur with involvement of the lateral condyle is quite rare. Several aspects make this particular case special, including the rarity of the location (lateral condyle of the distal femur), two successful joint-preserving surgeries on the patient suffering from primary and recurrent tumor by the same method, and long-term radiographic follow-up (12 years after revision) that established cement longevity and disease-free period.

Extended intralesional curettage with adjuvant chemical substances (phenol, hydrogen peroxide) and PMMA bone cementation represent well-known treatment choices for GCTB at accessible locations [1,2]. Cytotoxic effect of exothermic polymerization (60-80°) of PMMA occurs due to creation of an additional cytotoxic zone at the margins of the cavity during mechanical curettage [3]. In addition to the cytotoxic effect, bone cementing gives biomechanical stability of the defect, which is especially needed when subchondral plate integrity is doubtful, as well as radiological lucency for early detection of recurrence (unlike bone grafting) [4].

In the Behera et al. series of 22 patients with recurrent GCTB about the knee joint between 2009 and 2022, being one of the largest series from India, re-extended curettage along with filling either with bone grafting or PMMA was performed in 17 cases with results comparable to those in primary surgeries [7]. This is in agreement with our case, where the tumor developed after many years with an extended period of follow-up. It is interesting to note that the current 2014 recurrence occurred within the classic two-year period described in the literature [6,7], most probably due to the presence of Grade I-II histology along with CT-proven cortical and soft-tissue involvement, which are known to cause recurrence [7,8].

Management of recurrent GCTB continues to be controversial regarding re-curettage versus wide excision. Wide resection eliminates the risk of further local recurrence but mandates complex reconstruction typically with structural allograft or endoprosthetic replacement and carries risks of implant failure, periprosthetic fracture, and inferior long-term functional outcomes compared to joint-preserving techniques [9,10]. For recurrences that remain radiologically confined without joint contamination, pathological fracture, or extensive soft-tissue invasion, repeat extended curettage and bone cementing preserves the native joint and allows excellent functional recovery. The decision in our case to perform a repeat joint-preserving procedure was supported by the confined radiological appearance of the recurrence, the intact joint space, and the absence of pathological fracture. The subsequent 12-year disease-free course validates this surgical decision.

Denosumab, a RANK-L inhibitor, has been increasingly explored in GCTB management, particularly as a neoadjuvant to downstage large or surgically challenging lesions, or as adjuvant therapy in recurrent disease [9]. However, concerns regarding malignant transformation with long-term use, high cost, and the risk of recurrence upon discontinuation remain significant limitations. In our patient, the radiologically confined recurrence and the successful outcome of repeat surgery made denosumab unnecessary.

The serial radiographic follow-up in this case is particularly instructive. The 12-year follow-up documented progressive bone remodeling of the cortex around a stable cement mantle with no loosening, migration, or perilesional lucency at any timepoint. This demonstrates the long-term biocompatibility and structural durability of PMMA cement in the periarticular femoral environment.

At the recent follow-up, the patient demonstrated excellent functional outcome: full weight-bearing, knee flexion to approximately 120°, bilateral limb symmetry without deformity, and a well-healed scar. This is consistent with published long-term functional results for extended curettage and cementing in distal femoral GCTB, which report good-to-excellent functional outcomes in 80-90% of patients in large series [4,10].

## Conclusions

This case demonstrates that repeat extended curettage with phenolization and PMMA bone cementing is a viable, durable, and functionally superior limb-salvage strategy for radiologically confined recurrent GCTB of the lateral condyle of the distal femur. An exceptional 12-year disease-free outcome, full functional recovery, and cement integrity support this approach as the preferred management over extensive resection in carefully selected patients. Thorough adjuvant chemical curettage, adequate cement packing, and sustained long-term radiographic surveillance are the essential cornerstones of successful management in both primary and recurrent GCTB of the distal femur.
